# Supplementary material for: Representations of Research among Newly Graduated Paramedical Professionals: A Qualitative Study
Source: Int J Environ Res Public Health. 2021 Oct 28;18(21):11331. doi: 10.3390/ijerph182111331 (PMC8583123; doi:10.3390/ijerph182111331)
Supplement: Supplementary file 1 [file ijerph-18-11331-s001.zip › ijerph-1396232-supplementary.pdf]

Interview grid

Socio-demographic data

- Gender
- Age
- Paramedical profession
- Diploma obtained other than paramedical
- Highest degree obtained
- Place of work

Communication contract for the interview (explained orally before the interview)

First of all, I would like to thank you for agreeing to take part in this interview.

Let me introduce myself, I am Mathilde Chauliaguet, a Master's student in Education Sciences at the ISPEF Lyon 2. I am currently doing an internship in the Clinical Research and Epidemiology Department of the Hospices Civils de Lyon.

I am continuing the RECLIP 2 study in which you participated last year as a student by answering a questionnaire. Following this questionnaire, where you left us your contact details for an interview, we contacted the people who wished to take part in the interview and then drew lots according to profession among the people who still wished to take part in this interview.

This survey is about professionalisation in the paramedical field of rehabilitation. Therefore I would like to collect your views and professional experience.

First of all, I'm going to explain what I mean by "interview", which is an exchange around a subject. I'll ask you some broad questions, which you'll be able to respond to and explain your experiences and opinions on. There are no right or wrong answers, the interest is that you can express yourself as freely as possible. I am not trying to evaluate your practice or your knowledge. You can come back at any time during the interview to different points or elements that we have already discussed. You can decide not to answer certain questions and to stop the interview at any time.

The duration of the interview can normally be a short hour. This interview will take place in the respect of your confidentiality, your principles and your values. And I will be non-judgmental and benevolent.

For practical reasons and to facilitate transcription, this interview will be recorded. This recording will be heard exclusively by me for transcription and destroyed at the end of this research. In order to protect your anonymity, your name, the names you mention and the place you live in will be changed during the transcription.

Do you agree to have this interview recorded?

If you have any questions you can ask me, I will answer you immediately or at the end of the interview depending on the questions so as not to influence the rest of the interview.
Do you have any questions before we start?

Issue(s) explored	Question	Dunning
The place of research and evidence in professional practice	In your professional practice, when you have decisions to make or choices to make, what elements do you rely on? Your training? Your experience? The experiences of your colleagues? Other things?<br><br>In the course of your professional practice, have you ever had the opportunity to participate in or implement a research activity?<br>If so, give an example.	You mentioned research evidence in the elements you take into account. Can you tell me a bit more about that?<br>Why didn't you take into account the research evidence in making your decisions?<br><br>How do you use research data in your professional activity? Can you give me some examples?<br><br>How can you access this research data? Or do you think it is available? Are they easily accessible?<br><br>Is it easy to integrate research evidence into your practice? If so, why? If not, what might hinder this integration?<br><br>What are the obstacles and support points for setting up a research activity?<br><br>What means do you need to conduct a search?
The role of the context in the implementation of a research activity	In what context do you practice your profession? In your opinion, is it favorable for the implementation of research in the paramedical field? Why or why not?	In your opinion, would the institution in which you work look favorably on the implementation of a paramedical research activity? Why or why not?<br><br>Does the organisation in your workplace allow for research? Why or why not?<br><br>Would your superiors be in favor of implementing a research activity? And your colleagues? Why or why not?

		Are the institutions that represent you (trade unions, professional associations, ...) driving forces in the implementation of a research activity? If yes, how and why? If not, why not?
The place of research in professional identity	What do you think defines a good "xxxx"? What are their main characteristics or areas of expertise?	In your opinion, what place does research activity have in the professional identity of xxx?<br><br>To what extent could the initial training of xxx allow for more integration of research activity into the identity of XXX? How could this be done?<br><br>In your opinion, is it possible that in the future, xxx people could be more actively involved in the implementation of research that concerns them? Why or why not?
Improve and increase research and use of evidence in the allied health field	What would motivate you to use evidence and research in your profession?	How can you improve your studies to motivate you to do research and use evidence?
